# Editorial: Transcription Factors Drivers of Malignancy, Progression and Poor Outcomes in Breast Cancer

**DOI:** 10.3389/fonc.2022.912542

**Published:** 2022-06-27

**Authors:** Eva Parisi, Marta Marqués, Mariona Pont, Maria Alba Sorolla, Anabel Sorolla

**Affiliations:** Research Group of Cancer Biomarkers, Lleida Institute for Biomedical Research Dr. Pifarré Foundation (IRBLleida), Lleida, Spain

**Keywords:** transcription factors, breast cancer, metastasis, miRNAs, lncRNAs, prognosis, bioinformatics

In recent years, several unknown transcription factors (TFs) and associated regulatory molecules including miRNAs and lncRNAs, have been found to play a role in the initiation and progression of breast cancer (BC). However, only in a few cases, the signaling mechanism has been unraveled. Moreover, some research has been performed in order to delineate the potential of TFs as prognosis predictors in BC. This is particularly interesting since a significant proportion of oncoproteins are TFs and not much investigation has been done in this area. In this topic, we will highlight some significant discoveries involving TFs focusing on their role in tumorigenesis and in the prediction of clinical outcome in BC (**Figure 1**).

**Figure 1:**
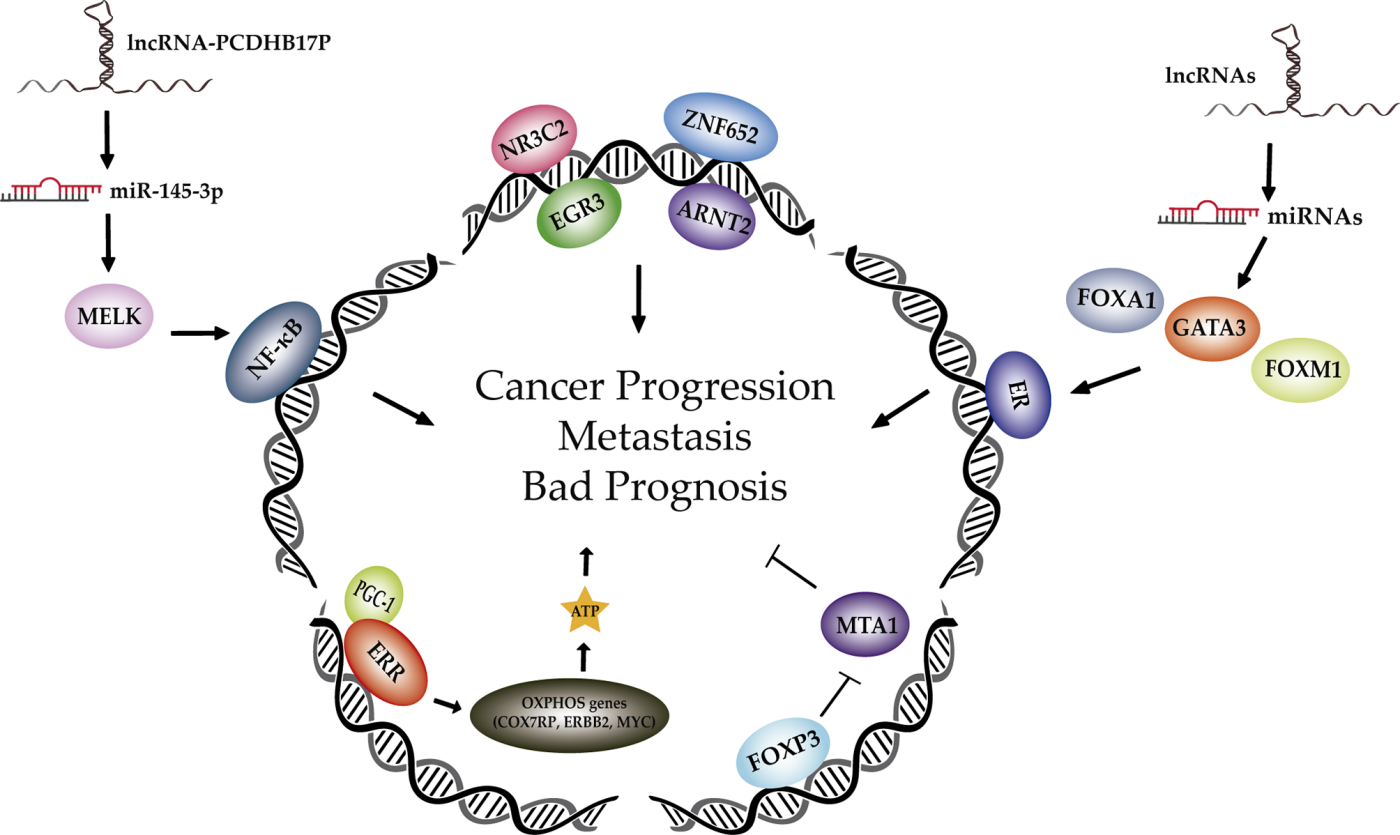
Different transcription factors and associated regulatory members involved in breast cancer progression and metastasis. Schematic representation on recent advances in the discovery of new TF, miRNAs and lncRNAs implicated in BC tumorigenesis. Known TFs involved in BC tumorigenesis are in grey while the emerging ones explained in this topic appear in colour. Abbreviations: ribonucleic acid (RNA), microRNA (miRNA), long non-coding RNA (lncRNA), maternal embryonic leucine zipper kinase (MELK), nuclear factor kappa B (NF-kB), nuclear receptor subfamily 3 group C member 2 (NR3C2), zinc finger protein 652 (ZNF652), early growth response 3 (EGR3), aryl hydrocarbon receptor nuclear translocator 2 (ARNT2), estrogen receptor (ESR), forkhead box A1 (FOXA1), GATA binding protein 3 (GATA3), forkhead box M1 (FOXM1), forkhead box P3 (FOXP3), metastasis-associated 1 (MTA1), estrogen related receptor (ERR), peroxisome proliferator-activated receptor-g coactivator-1 (PGC-1), oxidative phosphorylation (OXPHOS), cytochrome c oxidase subunit 7a related polypeptide (COX7RP), receptor tyrosine kinaseerbB 2 (ERBB2).

New drivers of BC tumorigenesis controlled by TFs have recently been discovered. One of them is cytochrome c oxidase subunit 7a related polypeptide (COX7RP). Kamada et al. found that COX7RP is overexpressed in BC tissues and correlates with patients’ poor survival. Interestingly, COX7RP has a role in promoting mitochondria respiratory supercomplex assembly and glutamine metabolism. COX7RP was initially discovered as an estrogen responsive gene. Later, the TFs estrogen related receptor (ERR) and the coactivator peroxisome proliferator-activated receptor-γ coactivator-1 (PGC-1) were found to be involved in the upregulation of mitochondrial oxidative phosphorylation (OXPHOS) genes, including COX7RP. The activation of the ERR/PGC-1s axis ensures the coverage of metabolic requirements in normal tissue. In contrast, in cancer cells, promotes progression and metastasis thereby increasing mitochondrial biogenesis, ATP production and the activation of oncogenic genes such as receptor tyrosine kinase 2 (ERBB2) and MYC. Targeting COX7RP, and consequently the respiratory supercomplex assembly, represents an interesting strategy to explore in BC, taking into account that the mitochondrial disassembly promoted by chemical agents has been proved to suppress tumor growth and metastasis in BC cells (Kamada et al., 2022).

The discovery of new prognosis biomarkers is essential for BC research. In this regard, by comparing differential gene expression with its corresponding progression free survival (PFS), Liu et al. have found that the expression of four TFs, nuclear receptor subfamily 3 group C member 2 (NR3C2), zinc finger protein 652 (ZNF652), early growth response 3 (EGR3) and aryl hydrocarbon receptor nuclear translocator 2 (ARNT2) (Liu et al., 2021), could discern a high-risk group of BCs characterized by shorter PFS. Interestingly, such a group was associated with enhanced mammalian target of rapamycin complex_1_ (mTORC_1_) signaling, suggesting a role of this signaling pathway in the pathogenesis of BC. To achieve this, they employed public data sets from The Cancer Genome Atlas (TCGA) and the Gene Expression Omnibus (GEO) repository, performed weighted gene co-expression network, function enrichment and Cox regression analyses.

Also, by deploying Omnibus data and performing bioinformatics analysis, Hassani et al. found five long non-coding RNAs (lncRNAs): APTR, AC144450.1, linc00663, ZNF337.AS1, and RAMP2.AS1; which were related to the forkhead box M1 (FOXM1)/GATA binding protein 3 (GATA3)/forkhead box A1 (FOXA1)/estrogen receptor 1 (ESR1) axis, when looking at lncRNAs altering the expression of such TFs belonging to the estrogen receptor (ESR) signaling. In recent years, the importance of lncRNAs in the regulation of gene expression has been proved. It is important pointing out that ESR expression is an indicator of BC patients’ prognosis and that ER acts as a TF modifying the expression of several genes. Its activity is influenced by cofactors such as GATA3, FOXA1 and FOXM1. After the validation of their expressions in clinical samples, the authors showed that GATA3 expression was significantly associated with the BC stage, FOXA1 and RAMP2.AS1 expressions with the mitotic rate, and FOXM1 and ZNF337.AS1 with breast feeding duration. Moreover, based on ROC curve analysis, the lncRNA AC144450.1 had the better diagnostic power to differentiate between cancerous and non-cancerous tissues (Hassani et al., 2021).

Another study also involving lncRNAs was performed by Zhu et al. In this work they identified the lncRNA PCDHB17P being upregulated in human BC tissues compared with the adjacent normal ones. They found a positive correlation between PCDHB17P and age or lymph node metastasis. PCDHB17P is located in the cytoplasm and plays a role in BC development, angiogenesis and metastasis through the modulation of epithelial-to-mesenchymal transition (EMT), cell proliferation, invasion and migration. In BC tissues, PCDHB17P could negatively regulate the expression of miR-145-3p, which has been significantly associated with a higher rate of overall survival. Further explorations demonstrate that PCDHB17P is positively correlated with maternal embryonic leucine zipper kinase (MELK) expression, a downstream target which expression is increased in BC. On the contrary, miR-145-3p suppresses MELK expression both at mRNA and protein levels. It has also been suggested that in BC lines, MELK might activate nuclear factor kappa B (NF-kB), which in turn, could regulate PCBHB17P by binding to its promoter and therefore, generating a feedback loop.

Apart from TFs, cofactors and lncRNAs promoting tumorigenesis, there are TFs positively associated with good prognosis in BC. It is the case of forkhead box P3 (FOXP3), a TF which has been shown to act as tumour suppressor by constraining metastasis through a mechanism not yet fully elucidated. By different bioinformatics analyses, the authors of this study identified downstream FOXP3 molecules involved in cell adhesion and cytoskeleton reorganization such as MTA1. Combining *in vitro* cell assays results, *in vivo* animal experiments and the study of clinical BC samples, they have confirmed that *MTA1* expression is regulated by promoter binding of FOXP3. Moreover, they have demonstrated a significant negative correlation between FOXP3 and MTA1 levels in BC. Therefore, this study provides new insights about the mechanisms by which FOXP3 inhibits BC metastasis (Liu et al.).

In summary, there have been notable advances in the discovery of novel drivers of BC tumorigenesis, among them, TFs. However, such a research has not yet been fully consolidated. Not surprisingly, some TFs have been considered good prognostic makers in BC. TFs are crucial proteins controlling either negatively or positively all cancer hallmarks. For this reason, the search of novel TFs indicators of clinical outcome is needed in order to predict BC prognosis more accurately.

## Author Contributions

AS assembled the editorial team, coordinated the work and wrote part of the manuscript. EP elaborated the figure and wrote part of the manuscript. MM, MP and MS wrote part of the manuscript. All the authors proof read the manuscript and approved the submitted version.

## Funding

This work is funded by the Instituto de Salud Carlos III (Spanish Health Ministry) with a Miguel Servet fellowship (CP20/0039) and the FIS Project PI21/00438 which are co-funded by the European Social Fund (ESF) “Investing in your future” and the European Union; the Emergent Re-search Group Recognition Award from the University and Research Grants Management Agency of Catalonia (Spain) under Grant 2017SRG1620. The research was supported by CERCA Programme of Generalitat de Catalunya and the IRBLleida—Fundació Dr. Pifarré.

## Conflict of Interest

The authors declare that the research was conducted in the absence of any commercial or financial relationships that could be construed as a potential conflict of interest.

## Publisher’s Note

All claims expressed in this article are solely those of the authors and do not necessarily represent those of their affiliated organizations, or those of the publisher, the editors and the reviewers. Any product that may be evaluated in this article, or claim that may be made by its manufacturer, is not guaranteed or endorsed by the publisher.

